# Aggrelyte‐2 promotes protein solubility and decreases lens stiffness through lysine acetylation and disulfide reduction: Implications for treating presbyopia

**DOI:** 10.1111/acel.13797

**Published:** 2023-02-23

**Authors:** Sudipta Panja, Rooban B. Nahomi, Johanna Rankenberg, Cole R. Michel, Hanmant Gaikwad, Mi‐Hyun Nam, Ram H. Nagaraj

**Affiliations:** ^1^ Department of Ophthalmology, School of Medicine, Sue Anschutz‐Rodgers Eye Center University of Colorado Anschutz Medical Campus Colorado Aurora USA; ^2^ Department of Pharmaceutical Sciences, Skaggs School of Pharmacy and Pharmaceutical Sciences University of Colorado Anschutz Medical Campus Aurora Colorado USA

**Keywords:** acetylation, disulfides, lens, *N* ^ε^‐acetyllysine, presbyopia, stiffness

## Abstract

Aging proteins in the lens become increasingly aggregated and insoluble, contributing to presbyopia. In this study, we investigated the ability of aggrelyte‐2 (N,S‐diacetyl‐L‐cysteine methyl ester) to reverse the water insolubility of aged human lens proteins and to decrease stiffness in cultured human and mouse lenses. Water‐insoluble proteins (WI) of aged human lenses (65–75 years) were incubated with aggrelyte‐2 (500 μM) for 24 or 48 h. A control compound that lacked the S‐acetyl group (aggrelyte‐2C) was also tested. We observed 19%–30% solubility of WI upon treatment with aggrelyte‐2. Aggrelyte‐2C also increased protein solubility, but its effect was approximately 1.4‐fold lower than that of aggrelyte‐2. The protein thiol contents were 1.9‐ to 4.9‐fold higher in the aggrelyte‐2‐ and aggrelyte‐2C‐treated samples than in the untreated samples. The LC–MS/MS results showed *N* ^*ε*^‐acetyllysine (AcK) levels of 1.5 to 2.1 nmol/mg protein and 0.6 to 0.9 nmol/mg protein in the aggrelyte‐2‐ and aggrelyte‐2C‐treated samples. Mouse (C57BL/6J) lenses (incubated for 24 h) and human lenses (incubated for 72 h) with 1.0 mM aggrelyte‐2 showed significant decreases in stiffness with simultaneous increases in soluble proteins (human lenses) and protein‐AcK levels, and such changes were not observed in aggrelyte‐2C‐treated lenses. Mass spectrometry of the solubilized protein revealed AcK in all crystallins, but more was observed in α‐crystallins. These results suggest that aggrelyte‐2 increases protein solubility and decreases lens stiffness through acetylation and disulfide reduction. Aggrelyte‐2 might be useful in treating presbyopia in humans.

## Graphical abstract

Presbyopia is a major vision‐impeding problem for many people over 40. Lens hardening (stiffening), due to protein insolubilization and aggregation, significantly contributes to presbyopia. Aggrelyte‐2 solubilizes aggregated proteins and reduces lens stiffness through lysine acetylation and disulfide reduction.

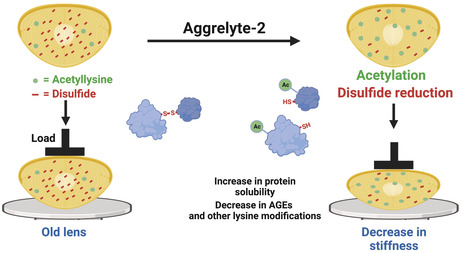

**Abbreviations**
AcK: *N* ^*ε*^‐acetyllysine
AGEs: advanced glycation endproducts
CEL: *N* ^*ε*^‐carboxyethyllysine
CML: *N* ^*ε*^‐carboxymethyllysine
FDR: false discovery rate
GSH: reduced glutathione
LACE: choline ester of lipoic acid
WI: water‐insoluble protein
WS: water‐soluble protein
αAC: αA‐crystallin
αBC: αB‐crystallin
βC: β‐crystallin
γC: γ‐crystallin
γDC: γD‐crystallin

## 1 INTRODUCTION

Presbyopia is a major vision‐impeding problem for many people over 40 years of age (Katz et al., 2021). Significant indicators of presbyopia are blurred vision at average reading distance, headaches, fatigue, and the requirement of reading glasses for vision correction. The estimates in 2015 were that there were approximately 1.8 billion people globally with presbyopia, and among them, nearly 26 million had visual impairment (Fricke et al., 2018). Presbyopia causes enormous productivity loss due to uncorrected vision, estimated at approximately 25 billion dollars a year (Berdahl et al., 2020). Thus, the development of methods for reversing, preventing, or postponing the onset of presbyopia is urgently needed.

Lens proteins have little turnover and therefore accumulate many posttranslational modifications (PTMs) throughout life. These include deamidated asparagine and glutamine residues (Hains & Truscott, 2010; Harrington et al., 2004; Sharma & Santhoshkumar, 2009) and advanced glycation end products (AGEs) formed from glycation (Fan & Monnier, 2021; Nagaraj, Linetsky, & Stitt, 2012). Other PTMs include proteolytically cleaved peptides, intra‐ and interprotein disulfides, kynurenine modifications, and racemized D‐aspartic acid (Garner & Spector, 1978; Hariharapura et al., 2013; Vazquez et al., 2002; Yu et al., 1985). The cumulative effects of PTMs over several decades lead to protein crosslinking, aggregation, and eventually insolubilization. The content of water‐insoluble protein (WI) in human lenses progressively increases up to ~40 years and then increases rapidly before plateauing later in life (Heys et al., 2007; Truscott & Friedrich, 2016).

Presbyopia results in the loss of the ability of the human lens to accommodate. One theory posits that changes in the stiffness gradient are responsible for the loss of accommodation (Weeber & van der Heijde, 2007). Another theory suggests that the stiffness of the lens nucleus is a significant determinant of presbyopia (Heys et al., 2004). However, it is generally accepted that age‐associated hardening of the lens is the principal cause of presbyopia (Heys et al., 2004; Petrash, 2013). It is believed that the accumulation of aggregated insoluble proteins leads to hardening and stiffening of the lens, especially in the central nuclear region of the lens, where proteins are older than in the outer lens cortex (Al‐Ghoul et al., 2001; Schachar & Pierscionek, 2007). The cumulative damage to proteins through the PTMs listed above appears to play a significant role in lens stiffening. Exposure to elevated temperature causing protein damage in the lens has also been implicated as a mechanism (Heys et al., 2007). In addition, age‐related thickening of the lens capsule (Barraquer et al., 2006; Krag et al., 1997), possibly due to the accumulation of PTMs in its proteins, and changes in the zonules that attach the lens to the ciliary body could also contribute to presbyopia.

The acylation of lysine residues in proteins is a common modification in proteins (Drazic et al., 2016). Acylation occurs from various acyl donors. Among the types of acylation, acetylation and succinylation are particularly well studied. In nuclear histones, acetylation is mediated by histone acetyltransferases and acetyl‐CoA, and its reversal by histone deacetylases regulates gene expression (Feron, 2019; Legube & Trouche, 2003). The acetylation of cytoplasmic proteins occurs through lysine aminotransferase or nonenzymatic reactions; in both reactions, acetyl‐CoA donates the acetyl group to lysine residues in proteins (Drazic et al., 2016; Wagner & Hirschey, 2014). Mitochondrial proteins are generally acetylated at a higher rate than cytoplasmic proteins due to higher acetyl‐CoA levels and slightly basic pH, which promotes nonenzymatic acetylation (Baeza et al., 2016; Wagner & Payne, 2013). The acetylation of mitochondrial and cytoplasmic proteins can alter cell metabolism (Baeza et al., 2016; Zhao et al., 2010).

In human lens proteins, lysine acetylation, succinylation, malonylation, and propionylation have been detected (Nagaraj, Nahomi, et al., 2012; Nahomi et al., 2020; Nandi et al., 2019). Among these modifications, *N* ^ε^‐acetyllysine (AcK) modification is the dominant modification (Nandi et al., 2019). The AcK levels in human lenses range from 1400 to 6200 pmol/mg protein, making it one of the major post‐translational modifications (PTMs) in lens proteins (Nandi et al., 2019). These levels are comparable to the levels of AGEs (Nagaraj, Linetsky, & Stitt, 2012), but lower than the isoaspartate levels (Warmack et al., 2019). Acetylation has been reported to induce structural alterations, increase lens protein stability, and improve α‐crystallin's chaperone activity (Moafian et al., 2016). We previously showed that AcK formation or the introduction of an AcK mimic at the K92C position increases the chaperone activity of αB‐crystallin (Nahomi et al., 2013). Further, acetylation of γD‐crystallin alters its interaction with α‐crystallin (DiMauro et al., 2014). In our initial studies, we made a serendipitous discovery that the introduction of AcK into lens proteins increased their water solubility. This finding led us to screen several compounds that can potentially donate an acetyl group to lysine residues in proteins as a means to solubilize aggregated proteins in aged lenses. Aggrelyte‐2 was among the best of several compounds tested for protein acetylation. In addition, we reasoned that aggrelyte‐2 could break disulfide bonds in lens proteins from the free –SH group after the donation of the acetyl group. Based on these characteristics, we anticipated that aggrelyte‐2 could both acetylate and reduce disulfide bonds and, through this dual action, solubilize aggregated lens proteins and decrease lens stiffness. This study presents evidence showing that aggrelyte‐2 solubilizes aggregated proteins in aged human lenses and reduces stiffness in mouse and human lenses.

## 2 RESULTS

### 2.1 Stability of aggrelytes under physiological conditions

**FIGURE 1:**
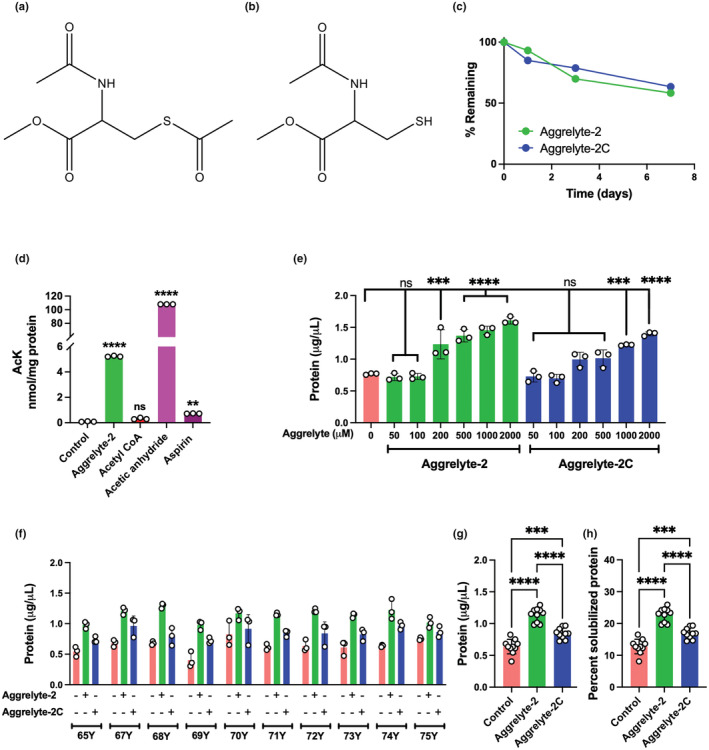
Aggrelytes solubilize human lens water‐insoluble protein (WI). Structure of aggrelyte‐2 (N,S‐diacetyl cysteine methyl ester) (a) and aggrelyte‐2C (N‐acetyl cysteine methyl ester) (b). Aggrelytes (each 2 mg/mL) were incubated in 50 mM phosphate buffer, pH 7.4, for 7 days at 37°C. NMR spectroscopy was used to determine stability (c). Comparison of the acetylating ability of aggrelyte‐2 and other acetylating agents (d); human recombinant αAC (3 mg/mL PBS) was incubated for 24 h with one of the following acetylating agents at 500 μM: aggrelyte‐2, acetyl‐CoA, acetic anhydride, or aspirin as described in the Section 4. The enzyme‐digested samples were analyzed for AcK by LC–MS/MS. WI (2 mg/0.4 mL) from a human lens (71 years) was suspended in 50 mM phosphate buffer, pH 7.4. To the suspension, aggrelyte‐2 or aggrelyte‐2C (0–2000 μM) was added and incubated for 24 h at 37°C (e); the suspension was centrifuged at 20,000 *g* for 20 min, and the soluble protein content in the supernatant was measured. At each concentration, each sample was separately processed three times and analyzed (mean ± SD). Statistical comparisons between the controls and treated samples were performed. WI (2 mg/0.4 mL) from aged lenses (65–75 years) was suspended in 50 mM phosphate buffer, pH 7.4, and treated with 500 μM of one of the aggrelytes for 24 h (f). Each sample was separately processed three times and analyzed (mean ± SD). The combined effects of the aggrelytes (from 10 lenses) on the solubilization of WI protein are shown in Panel ‘g’. The percent solubilized protein from the initial weight of WI after 24 h treatments is shown in Panel ‘h’. **p < 0.01, ***p < 0.001, ****p < 0.0001, ns, not significant.

The structures of aggrelyte‐2 and‐2C are shown in Figure 1a,b. The NMR spectral data (data not shown) indicated that the aggrelytes were relatively stable for 24 h but degraded by 30% and 42% (aggrelyte‐2) and 21% and 37% (aggrelyte‐2C) during the 3‐ and 7‐day incubation periods, respectively (Figure 1c, Table S1).

### 2.2 Protein acetylation capability of aggrelyte‐2

The relative ability of aggrelyte‐2 to acetylate lysine residues in proteins was investigated in αA‐crystallin (αAC) incubated in 50 mM sodium phosphate buffer, pH 7.4, with several acetylating agents at a 500 μM concentration. Acetic anhydride was the most potent acetylating agent (~21‐fold higher than aggrelyte‐2), followed by aggrelyte‐2, aspirin, and acetyl‐CoA (Figure 1d). We note that the near‐neutral pH used for incubation may have reduced the acetylating capacity of acetyl‐CoA.

### 2.3 Aggrelyte‐2 solubilizes WI of aged human lenses

We investigated the ability of aggrelytes to solubilize WI from a 71‐year‐old human lens by incubating WI (2 mg/0.4 mL) in 50 mM sodium phosphate buffer, pH 7.4, at 37°C with 50–2000 μM aggrelytes for 24 or 48 h. Our results at 24 h (Figure 1e) and 48 h (Figure S1) showed a progressive increase in the solubility of WI with increasing concentrations of aggrelyte‐2. Aggrelyte‐2C also increased the protein solubility, but the solubility was ~1.4‐fold inferior (at 500 μM) to that of aggrelyte‐2 at 48 h. Based on these results, we used aggrelytes at a concentration of 500 μM in all subsequent in vitro experiments.

Incubation of WI from aged lenses (65–75 years) at pH 7.4 and 37°C for 24 h yielded 0.40–0.83 μg protein/μL in the supernatant (Figure 1f,g), which corresponds to 8%–17% of the initial WI taken (Figure 1h). However, upon incubation with aggrelyte‐2, the yield increased to 0.97–1.30 μg/μL in the supernatants (Figure 1f,g), reaching 20%–26% of the initial protein (Figure 1h). Under similar conditions, aggrelyte‐2C yielded 0.72–0.97 μg/μL protein (Figure 1f,g), corresponding to 14%–19% of the initial WI (Figure 1h). The incubation of WI without aggrelytes for 48 h slightly increased the soluble protein in the supernatant to a yield of 0.48–1.01 μg protein/μL (Figure S2A,B), corresponding to 10%–20% of the initial protein (Figure S2C). The incubation with aggrelyte‐2 and aggrelyte‐2C yielded 0.96–1.48 μg/μL and 0.68–1.12 μg/μL protein in the supernatants (Figure S2A,B), corresponding to 19%–30% and 14%–22% of the initial protein (Figure S2C), respectively. Plots of the data from all samples indicate that aggrelyte‐2 was significantly (*p* < 0.001) ~1.4‐fold better than aggrelyte‐2C in solubilizing WI in the samples incubated 24 h and 48 h (the combined data are shown in panels Figure 1g and Figure S2B). Together, these results indicate a slight increase in the soluble protein yield in the samples incubated for 48 h compared to those incubated for 24 h, and aggrelyte‐2 was better than aggrelyte‐2C in solubilizing WI. The possibility of the release of water‐soluble proteins loosely adhering to WI upon aggrelyte‐2 treatment was tested. The lens from a 67‐year‐old donor was processed to obtain WI as above. WI (2 mg/0.4 mL) was suspended in 1 mL PBS and stirred for 2 min at RT. The suspension was centrifuged at 20,000 *g* for 20 min at 4°C. The resulting pellet was suspended again in 1 mL PBS, stirred at RT as before, and centrifuged. These steps were repeated four times. The resulting WI was lyophilized. The lyophilized WI was treated with 500 μM aggrelytes, and the solubilized protein was measured as above. The aggrelyte‐2‐ and aggrelyte‐2C‐treated samples showed 55% and 39% increases in water‐soluble proteins over the buffer‐treated control sample (Figure S3). The results also ruled out the release of water‐soluble proteins adhering to WI upon aggrelyte treatment.

To verify whether protein acetylation alone contributed to lens protein solubility, we compared the soluble protein levels in WI treated with acetic anhydride and aggrelyte‐2. Our results showed that aggrelyte‐2 was 1.3 times better than acetic anhydride in solubilizing WI, even though acetic anhydride was a better acetylating agent (Figure S4).

### 2.4 Higher AcK content in aggrelyte‐2 solubilized proteins

**FIGURE 2:**
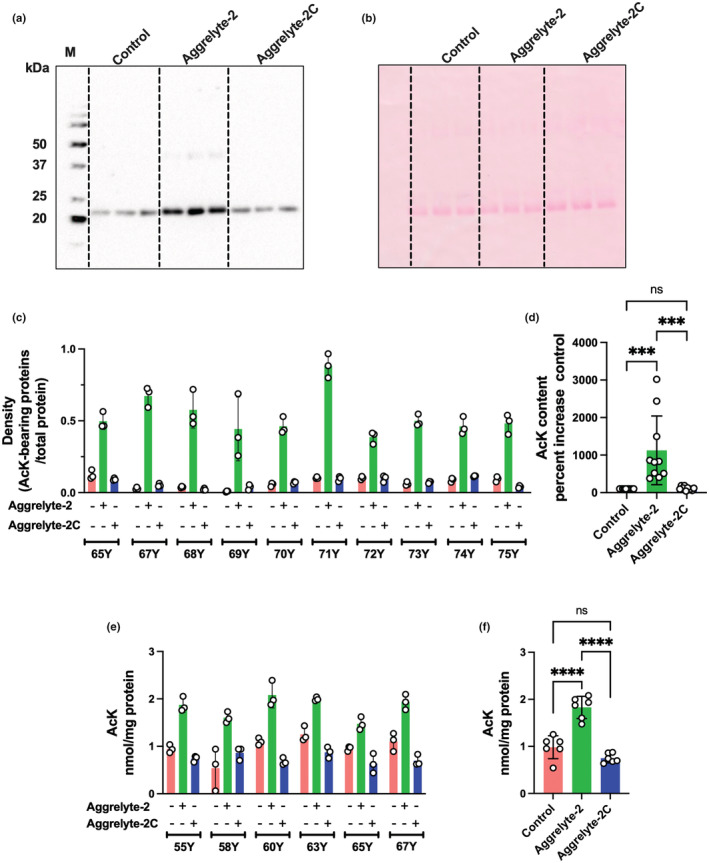
Aggrelyte‐2 acetylates proteins during the solubilization of water‐insoluble protein (WI). (a) A representative western blot of AcK is shown for WI from a 71‐year‐old lens treated with either aggrelyte‐2 or aggrelyte‐2C (each 500 μM) as described in Figure 1. (b) The Ponceau S‐stained membrane shows equal protein loading. Each sample was separately processed three times and analyzed, and the bar graphs represent the mean ± SD. A densitometric plot from the western blot analysis of AcK‐bearing proteins in 10 human lenses (65–75 years) is shown in ‘c’. The percent increase in the AcK content in the solubilized protein content over the control (no aggrelyte) in all lenses (total of 10, mean ± SD) is shown in ‘d’. The solubilized protein obtained from the treatment of WI from human lenses (55–67 years) with aggrelytes was digested with a series of enzymes and analyzed by LC‐/MS as described in Section 4 (e). Each sample was separately processed three times and analyzed, and the bar graphs represent the mean ± SD. A combined plot of the mean values of the three analyses of each sample is shown in ‘f’.  ***p < 0.001, ****p < 0.0001, M, molecular weight markers; ns, not significant.

We determined the content of AcK‐bearing proteins in the solubilized proteins from the above experiments by western blot analysis. Figure 2a shows a representative western blot of proteins from a 71‐year‐old lens. The Ponceau S‐stained membrane (Figure 2b) showed equal protein loading of the samples. We observed faint immunoreactivity for AcK in the control samples (not incubated with either aggrelyte) and the samples incubated with aggrelyte‐2C. This represented AcK formed in situ, an observation similar to that in our previous study (Nagaraj, Nahomi, et al., 2012). However, upon incubation with aggrelyte‐2, the amount of AcK‐bearing proteins increased, especially near the 20 kDa region, possibly indicating AcK modification in crystallins. In contrast to aggrelyte‐2, aggrelyte‐2C did not increase the AcK levels. Further analysis of 10 lenses (65–75 years old) by western blot analyses followed by densitometry indicated that aggrelyte‐2 significantly (*p* < 0.001) increased the amount of AcK‐bearing proteins relative to the samples treated with aggrelyte‐2C or untreated controls (Figure 2c,d).

LC–MS/MS analyses of the solubilized protein samples revealed 0.5–1.3 nmol of AcK/mg protein in the controls (Figure 2e). However, the aggrelyte‐2‐treated samples contained 1.5–2.1 nmol/mg protein. The levels in the aggrelyte‐2C‐treated samples were comparable to those in the controls (0.6–0.9 nmol/mg protein). When we plotted all data together, we observed a significant (*p* < 0.0001) 1.9‐ and 2.4‐fold increase in the AcK levels in the aggrelyte‐2‐treated samples compared to the controls and aggrelyte‐2C‐treated samples (Figure 2f).

### 2.5 Individual crystallin levels in the solubilized human lens protein

**FIGURE 3:**
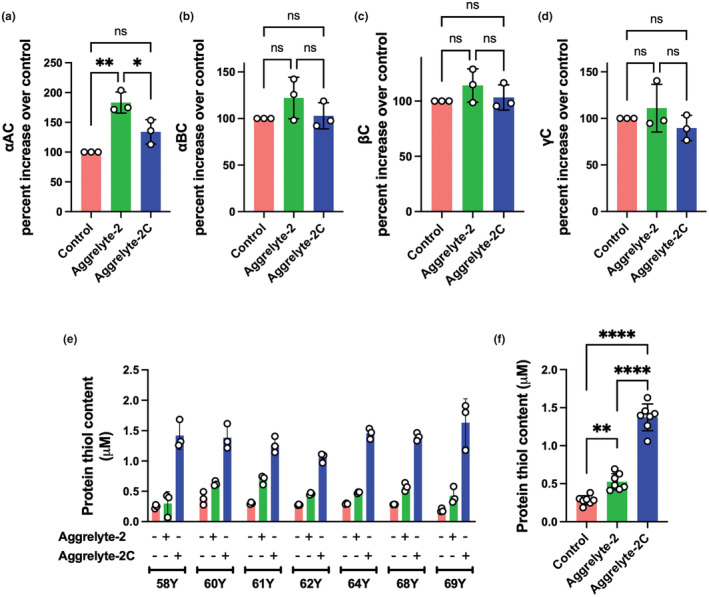
Individual crystallin levels and protein‐thiol content in the solubilized protein. The major crystallin subtypes were measured by western blot analysis followed by densitometry in the solubilized WI of three lenses (68‐, 73‐, and 75‐year‐old lenses) treated with either aggrelyte‐2 or aggrelyte‐2C (500 μM). Other details are as described in Figure 1. αAC (a), αBC (b), βC (c), and γC (d). The bar graphs represent the mean ± SD of *n* = 3 measurements. The protein‐thiol content in the solubilized protein from WI from aged lenses (58–69 years old) was measured using a Thiol Quantification Assay Kit as described in Section 4 (e). The data are the mean ± SD for each sample processed three times separately and analyzed. A combined plot of the mean values of the three analyses of each sample is shown in ‘f’. *p < 0.05, **p < 0.01, ****p < 0.0001, ns, not significant.

We measured crystallins (αAC, αB‐crystallin [αBC], β‐crystallin [βC], and γ‐crystallin [γC]) in the aggrelyte‐solubilized protein (from WI) by western blot analyses (Figure S5). The levels of αAC were 1.8‐fold higher (*p* < 0.01) in the aggrelyte‐2‐treated samples than in the controls and 1.3‐fold higher (*p* < 0.05) than in the aggrelyte‐2C‐treated samples (Figure 3a). The levels of αBC were 1.2‐fold (not significant) higher in aggrelyte‐2‐treated samples than in the controls and 1.1‐fold higher (not significant) than in the aggrelyte‐2C‐treated samples (Figure 3b). The levels of βC and γC were slightly but not significantly higher in the aggrelyte‐2‐treated samples than in the controls. Aggrelyte‐2C had no effect on the levels of either βC or γC (Figure 3c,d).

### 2.6 Increased protein thiol content upon aggrelyte treatment

The protein‐thiol content in solubilized protein samples from WI of aged lenses (58–69 years) was determined using a Thiol Quantification Assay Kit. In the control samples, the thiol content was 0.2–0.4 μM (Figure 3e,f). However, in the aggrelyte‐2‐treated samples, the content was 0.3–0.7 μM. These values were significantly higher than those in the controls (*p* < 0.01). In the aggrelyte‐2C‐treated samples, the thiol levels were even higher (1.1–1.6 μM, 2.6‐fold) than those in aggrelyte‐2‐treated samples (*p* < 0.0001). Together, these results suggested that aggrelytes can reduce disulfide bonds in proteins.

### 2.7 Aggrelytes are not cytotoxic to lens epithelial cells

Before we investigated the ability of aggrelytes to reduce lens stiffness ex vivo, we tested whether they exerted toxic effects on lens epithelial cells. We did not observe a loss in cell viability up to 1000 μM of aggrelytes in either mouse (Figure S6A) and human lens epithelial cells (Figure S6B). In fact, it appeared that aggrelytes promoted cell growth in mouse lens epithelial cells (and in human lenses at low concentrations). We used 1000 μM aggrelytes in subsequent ex vivo experiments.

### 2.8 Aggrelyte‐2 reduced stiffness in mouse and human lenses and increased soluble protein content in human lenses

**FIGURE 4:**
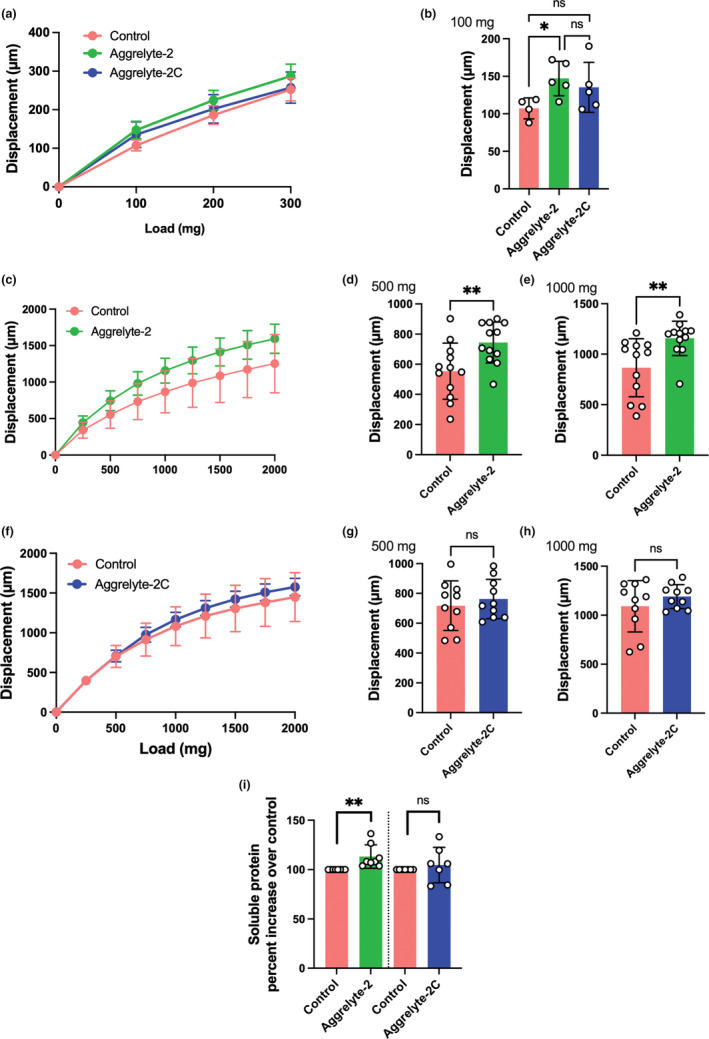
Aggrelyte‐2 reduces the stiffness of mouse and human lenses. Mouse lenses (from 6‐month‐old C57BL6/J mice, *n* = 4–5) were incubated without (control) or with aggrelyte‐2 or 2C (1 mM) for 24 h in serum‐free medium as described in Section 4. Freshly obtained human lenses (42–68 years old, *n* = 10–12) were incubated without (control) or with aggrelyte‐2 or 2C (1 mM) for a total of 72 h in serum‐free MEM with a change in media containing freshly dissolved aggrelytes every 24 h. Lens stiffness was measured as described in Section 4. The bar graph on the right for mouse lenses shows changes in displacement at a 100 mg load (a, b). The changes in displacement for human lenses treated with aggrelyte‐2 at loads of 500 and 1000 mg are shown in (c–e), and changes in displacement for human lenses treated with aggrelyte‐2C at loads of 500 and 1000 mg are shown in (f–h). The water‐soluble proteins content in aggrelyte‐treated human lenses (*n* = 7‐8) is shown in ‘i’. The controls in each case were contralateral lenses. The bar graphs represent the mean ± SD of measurements. *p < 0.05, **p < 0.01, ns, not significant.

Incubation of human lenses with aggrelytes did not alter the lens transparency, as revealed in four sets of lenses (age: 60 and 62 years) (Figure S7A,B is one set and Figure S7C,D is another); the transparency was comparable to the untreated contralateral control lenses after 72 h of incubation. Incubation of lenses for 72 h increased the weight of lenses by 2.9%–4.7% (Figure S7E). However, this increase in weight was independent of whether aggrelyte‐2 (Figure S7E) or aggrelyte‐2C (Figure S7F) was present or not in the medium. The stiffness of the lenses was measured after incubation with aggrelytes and compared to those of the untreated control lenses. The lens displacement (in μm) due to the load applied was used as the measure of lens stiffness; the softer the lens, the more displacement it exhibits. Aggrelyte‐2 reduced the stiffness in 6‐month‐old mouse lenses (the bar graph on the right is at 100 mg load) (Figure 4a,b). The effect was statistically significant (*p* < 0.05) compared to the controls. It was statistically insignificant when compared to the aggrelyte‐2C; the increase in displacement with aggrelyte‐2 was 8.7% greater when compared to the aggrelyte‐2C‐treated lenses. Furthermore, the results indicated that aggrelyte‐2 significantly (*p* < 0.05) reduced the axial strain of the mouse lenses compared to controls or aggrelyte‐2C‐treated lenses (at 100 mg load, Figure S8A). The aggrelyte‐2 treatment of human lenses (age: 42–68 years) showed significant increases in displacement (Figure 4c). The bar graphs on the right show the stiffness measured at loads of 500 and 1000 mg (Figure 4d,e). At both loads, aggrelyte‐2 significantly reduced the stiffness of lenses compared to the untreated controls (*p* < 0.01). However, aggrelyte‐2C did not show significant effects (Figure 4f–h). Aggrelyte‐2 treatment, but not aggrelyte‐2C significantly (*p* < 0.01) reduced the axial strain compared to controls at both 500 mg and 1000 mg loads (Figure S8B,C). Together, these results suggested that aggrelyte‐2 can reduce the stiffness of aged mouse and human lenses. The WS content was 13.2% higher (*p* < 0.01) in the aggrelyte‐2‐treated human lenses than in the untreated controls (contralateral lenses, Figure 4i), but the difference (4.6% higher) did not reach statistical significance when compared to the aggrelyte‐2C‐treated lenses. Together, these results suggested that aggrelyte‐2, but not aggrelyte‐2C, was able to reduce stiffness in mouse and human lenses.

**FIGURE 5:**
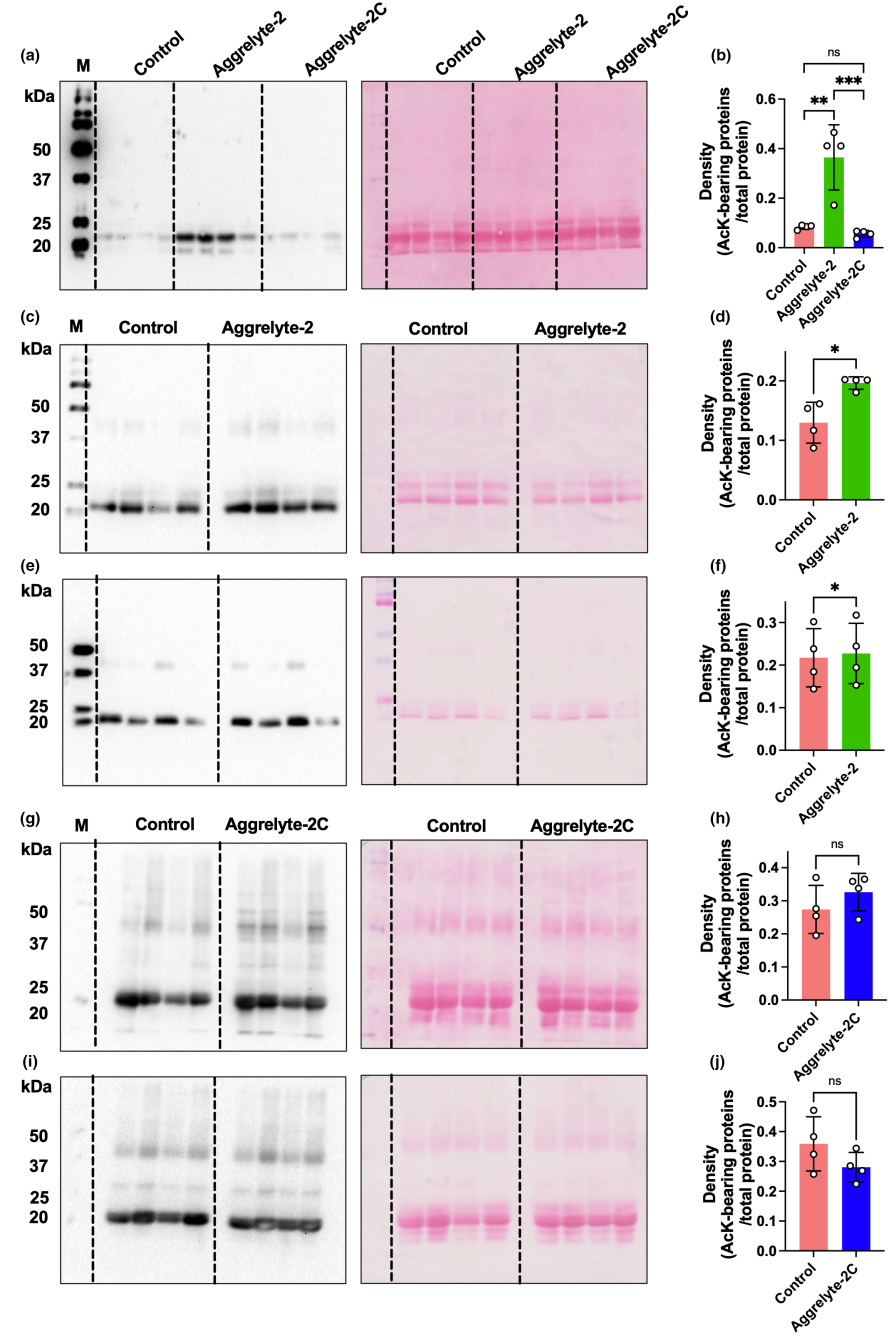
Treatment with aggrelyte‐2 increases the levels of AcK‐bearing proteins in mouse and human lenses. Lenses were incubated as described in Figure 4. The AcK‐bearing proteins in water‐soluble protein were measured by western blot analysis. Each sample was separately processed three times and analyzed. Ponceau S‐stained membranes showed equal protein loading. Aggrelyte‐2 increased the acetylation of proteins in mouse (a, b) and human cortical (c, d) and nuclear (e, f) proteins. However, treatment with aggrelyte‐2C had no effect on AcK levels in either cortical (g, h) or nuclear proteins (i, j). The bar graphs (densitometric plots) represent the mean ± SD of *n* = 4 measurements. *p < 0.05, **p < 0.01, ***p < 0.001. M, molecular weight marker, ns, not significant.

**FIGURE 6:**
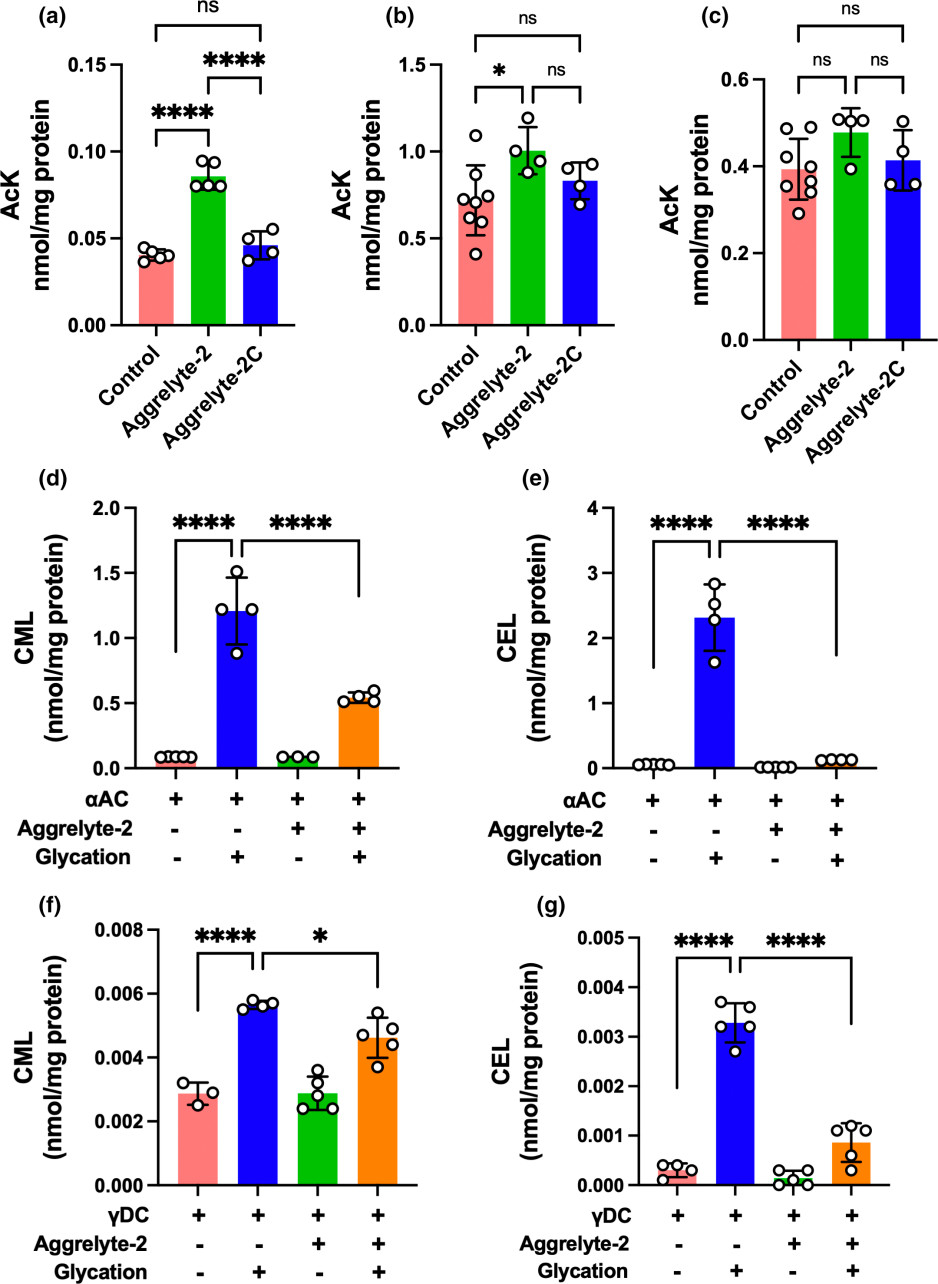
Aggrelyte‐2 increases the AcK levels in lenses and inhibits AGE formation in human lens proteins. Mouse (a) and human (b, c) lenses were treated with or without aggrelytes as described in Figure 4. WS from lenses (mouse) and proteins from the cortical (b) and nuclear (c) human lenses were digested with a series of enzymes and analyzed by LC–MS/MS as described in Section 4. The bar graphs represent the mean ± SD of measurements (*n* = 3‐5). Human recombinant αAC (3 mg/mL, d and e) or γDC (2 mg/mL, f and g) in PBS was treated with or without aggrelyte‐2 (10 mM for αAC and 1.25 mM for γDC) for 24 h, dialyzed and incubated with or without a glycating mixture for 5 days at 37°C. The incubated proteins were dialyzed and treated with 100 mM sodium borohydride and acid hydrolyzed, after which the *N* ^*ε*^‐carboxymethyllysine and *N* ^*ε*^‐carboxyethyllysine were analyzed by LC–MS/MS as described in Section 4. Each sample was separately processed 3–4 times. The bar graphs represent the mean ± SD of 3–4 measurements. *p < 0.05, ****p < 0.0001. ns, not significant.

Western blot analyses of the water‐soluble proteins (WS) of mouse lenses (Figure 5a) showed significantly higher AcK modifications in the aggrelyte‐2‐treated lenses than in the untreated controls (*p* < 0.01) and aggrelyte‐2C‐treated lenses (*p* < 0.001) (Figure 5b). Equal protein loading is confirmed by Ponceau‐S staining of the membrane. The aggrelyte‐treated and untreated human lenses were separated into cortical and nuclear proteins, and the AcK content was assessed by western blot analysis. The results showed significantly higher AcK content in both the cortical and nuclear proteins (*p* < 0.05) of aggrelyte‐2‐treated lenses than in the untreated lenses (Figure 5c–f). The AcK levels in the cortical and nuclear proteins of aggrelyte‐2C‐treated lenses were statistically insignificant when compared to those in the untreated controls (Figure 5g–j). In addition, the LC–MS/MS results showed significantly (*p* < 0.0001) higher AcK content in aggrelyte‐2‐treated mouse lenses than in the aggrelyte‐2C‐treated or untreated control lenses (Figure 6a). Similarly, human lens cortical proteins showed significantly (*p* < 0.05) higher levels of AcK when compared to the untreated control lenses (Figure 6b). However, in the case of nuclear proteins, there was a slight but statistically insignificant difference between the aggrelyte‐2‐treated lenses and controls or aggrelyte‐2‐treated and aggrelyte‐2C‐treated lenses (Figure 6c). The absence of a significant difference in the AcK levels in the nuclear proteins of aggrelyte‐2 treated lenses when compared to the controls in the LC–MS/MS analyses despite a significant difference between the two groups in western blotting data is possibly due to the poor enzymatic digestion of the highly cross‐linked nuclear proteins (prior to LC–MS/MS analyses). Nonetheless, these results suggested that aggrelyte‐2 permeated the lenses (cortex, and possibly nucleus in the case of the human lenses), acetylated lysine residues in proteins, and promoted the solubility of proteins.

### 2.9 Identification of AcK sites and the relative levels at each site

The AcK modification sites and relative levels at those sites in the aggrelyte‐2‐treated and untreated (control) lens cortical proteins were evaluated in the lens (from a 49‐year donor) that had the largest overall AcK differential measured by western blot analysis (Figure 5c) and LC–MS/MS (Figure 6b). Three hundred fifteen AcK‐bearing peptides that passed a 0.5% false discovery rate (FDR) filter were identified (Table S2), corresponding to 170 AcK sites and 59 proteins. Fourteen AcK‐sites, corresponding to 20 AcK peptides and 11 proteins, were identified in the aggrelyte‐2‐treated lens that was at least threefold greater than similar AcK sites in the untreated control lens (Table 1). Among those, six AcK sites from six proteins were at least 5‐fold greater than the peptides from untreated control lenses. AcK at K88 in αAC and K370 in the α1A‐chain of tubulin were not detected in the control lens but were prominent in the aggrelyte‐2‐treated lens. Both lens samples were spiked with 200 ng of acetylated bovine serum albumin (AcBSA) protein to ensure that fold change values for identified AcK sites were not due to the sample workup or instrument errors. Seventeen AcBSA peptides were identified in both treated and control lenses and, when summed, yielded a 1.05‐fold change in the treated vs. control lenses. There was very little change in the overall AcBSA signal, indicating that the AcK‐bearing peptide measurements are a true reflection of the acetylation levels in the treated and control lenses.

**TABLE 1:** Identification of AcK sites in proteins of the aggrelyte‐2 treated lens.

Protein name	Uniprot accession #	AcK site	Peptide count AcK site	Aggrelyte‐2 treated area	Control area	Fold change (Aggrelyte‐2 vs. control) [Total sum]
AlphaA‐crystallin	P02489	K88	1	1.11 E+05		*
Tubulin alpha‐1A chain	Q71U36	K370	1	4.24 E+04		*
BetaB2‐crystallin	P43320	K42	1	6.98 E+05	2.71 E+04	25.80
Phakinin	Q13515	K400	1	2.21 E+05	1.52 E+04	14.48
Retinal dehydrogenase 1	P00352	K495	1	9.11 E+04	9.99 E+03	9.12
AlphaB‐crystallin	P02511	K174	3	5.84 E+06	7.88 E+05	7.41
Filensin	Q12934	K360	1	1.16 E+05	2.94 E+04	3.94
Cytidine deaminase	P32320	K51	1	7.26 E+05	1.99 E+05	3.66
AlphaB‐crystallin	P02511	K72	1	6.31 E+05	1.77 E+05	3.56
Actin cytoplasmic 1	P60709	K213	1	1.03 E+05	2.92 E+04	3.53
Actin cytoplasmic 1	P60709	K61	1	7.63 E+04	2.19 E+04	3.48
Peroxiredoxin‐6	P30041	K56	2	5.83 E+05	1.79 E+05	3.26
Glyceraldehyde‐3‐phosphate dehydrogenase	P04406	K263	1	1.04 E+05	3.27 E+04	3.18
AlphaA‐crystallin	P02489	K70	4	8.80 E+06	2.81 E+06	3.13
Acetylated Bovine Serum Albumin Standard Spike at 200 ng in each sample	N/A	N/A	17	3.68 E+07	3.85 E+07	−1.05

*Note*: Aggrelyte‐2 treated and control paired lens water‐soluble proteins were tryptically digested and immunoprecipitated for AcK‐bearing peptides and analyzed by LC–MS/MS to identify AcK sites. Tandem mass spectra were then searched in PEAKS X Proteomics Studio against the Swissprot Homo sapiens database with variable oxidation (M), deamidation (NQ), and AcK and fixed carbamidomethylation (C) modifications. MS‐only level data were then extracted from the two data files using the formula, mass, and retention time from AcK‐bearing peptides identified in database searching. The areas of peptides identified with a specific AcK site were summed up to represent the total signal for that given AcK site, and the number of peptides is denoted in the peptide count AcK site column in the table. Fourteen AcK sites from 11 proteins passed a manually curated average fold‐change filter of three or more in the treated vs. untreated sample. The AcK at K88 in αA‐crystallin and AcK site K370 in tubulin alpha‐1A chain were only found in the aggrelyte‐2 treated sample (denoted with *). Additionally, 200 ng of acetylated BSA (AcBSA) standard was spiked into each sample at the beginning of sample preparation to control for variance in acetylation levels due to sample preparation. Seventeen AcK peptides were detected from the acetylated BSA standard. Peptide areas were summed corresponding to a 1.05‐fold change in the treated vs. control sample and therefore no normalization was needed.

### 2.10 Morphological changes in the lens upon aggrelyte‐2 treatment

We assessed morphological changes in human lenses upon aggrelyte treatment (1 mM, 72 h, assessed at 24 h intervals). Lens sections were stained with lectin‐FITC, and fluorescence images of the outer cortical region (proximal to the epithelial cell layer) were captured by fluorescence microscopy. No apparent changes were observed in the lens morphology of aggrelyte‐2‐ or ‐2C‐treated lenses when compared to the untreated control (Figure S9).

### 2.11 Effect of aggrelyte‐2 on AGE formation in lens proteins

We assessed the effect of aggrelyte‐2 on AGE formation in αAC and γD‐crystallin (γDC). Two AGEs, *N* ^*ε*^‐carboxymethyllysine (CML) and *N* ^*ε*^‐carboxyethyllysine (CEL), were measured by LC–MS/MS. The levels of both AGEs increased upon glycation (Figure 6d–g). However, the treatment of proteins with aggrelyte‐2 prior to glycation significantly reduced (*p* < 0.0001) the levels of both CML and CEL. These results suggested that aggrelyte‐2 inhibited AGE formation in lens proteins through AcK formation.

## 3 DISCUSSION

The objective of this study was to develop compounds that can promote the solubilization of aggregated proteins in the lens and thereby decrease the age‐associated stiffness of human lenses. Our results showed that aggrelytes partially solubilized WI proteins and reduced stiffness in cultured mouse and human lenses ex vivo. Aggrelyte‐2 was more effective than aggrelyte‐2C; this appears to be due to the ability of aggrelyte‐2 to acetylate lysine residues. Our results showed that the solubilized proteins obtained from the aggrelyte‐2 treatment (of WI from human lenses) contained higher levels of AcK than the untreated samples and those treated with aggrelyte‐2C.

The mass spectrometry data showed that AcK formation from aggrelyte‐2 occurs in crystallins, cytoskeletal proteins, and other lens proteins. α‐Crystallin appears to be one of the major targets for aggrelyte‐2‐mediated acetylation. βB2‐Crystallin is another prime target. The significance of preferential acetylation in these proteins to lens protein solubility and stiffness reduction could be investigated in future studies. Whether aggrelyte‐2 treatment altered cell signaling through acetylation of proteins in the lens epithelium also needs to be investigated.

The increase in the solubility of WI may occur as a result of combined modifications in these proteins or from a few specific proteins. Our study could not tease out the contribution(s) of specific modified protein(s) to WI solubility. How acetylation increases protein solubility is uncertain. Acetylation neutralizes the positive charge in lysine residues, which could interfere with the ability to form hydrogen bonds with negatively charged carboxylate groups in proteins. Furthermore, acetylation could disrupt the salt bridges that can be formed between the positively charged amino group in lysine residues and negatively charged amino acids such as glutamic acid. These disruptions could contribute to increasing protein solubility (Walker et al., 2021).

We expected similar protein thiol content after treatment with the two agents due to the reduction of disulfide bonds in proteins. However, the higher thiol content in the samples treated with aggrelyte‐2C compared to that in the samples treated with aggrelyte‐2 suggested that aggrelyte‐2 is weaker than aggrelyte‐2C in reducing protein disulfides. This could be because aggrelyte‐2 must donate the S‐acetyl group to lysine residues before being able to reduce disulfides.

While acetylation alone can increase protein solubility, as reported in plant proteins (Lawal & Adebowale, 2004; Miedzianka et al., 2021), we used aggrelyte‐2 to acetylate lysine residues and break disulfide bonds in proteins sequentially. Disulfide bond formation in proteins has been linked to the weakening of antioxidative capabilities in aging lenses (Taylor & Davies, 1987; Truscott & Friedrich, 2019). Disulfides are considered among the significant contributors to the aggregation of lens proteins during presbyopia and cataract formation (Garner & Garner, 2016). Garner and Garner (2016) developed lipoic acid choline ester (LACE) to target disulfide bonds in aging lens proteins. Treatment with LACE reduced lens stiffness in mouse and human lenses (Korenfeld et al., 2021). While targeting disulfides is strategically sound, alone, it may not disaggregate the bulk of the WI in the lens. Our data showed that despite the greater ability of aggrelyte‐2C to reduce protein disulfides, its ability to solubilize WI was significantly inferior to that of aggrelyte‐2, suggesting that protein disulfides are not the sole contributors to protein insolubility in human lenses.

The oral administration of resveratrol and lactic acid bacteria has also been found to reduce lens stiffness by targeting oxidative stress in rats (Nagashima et al., 2021). In addition, a randomized clinical trial of pirenoxine showed improvement in the accommodative ability of lenses in humans and elasticity in rat lenses exposed to cigarette smoke (Tsuneyoshi et al., 2017). However, the mechanisms by which pirenoxine causes these changes are unclear. Other presbyopia treatments that have been attempted include increasing the depth of the visual field by parasympathetic‐mediated miosis and ciliary muscle stimulation (Katz et al., 2021). Nevertheless, the effects of such treatments are usually transient. The ability of aggrelyte‐2 to solubilize protein through acetylation and disulfide reduction may be superior to that of other tested agents in terms of reducing stiffness in aging lenses.

While the preliminary efficacy clinical trial showed promising results for LACE (UNR844) (Garner & Garner, 2016), how long the effect persists in the lens following its topical application is unclear. It is possible that LACE‐mediated disulfide reduction can diminish over time due to the depletion of LACE from the lens and the continued exposure of lens proteins to oxidative stress. Thus, LACE may have to be applied periodically to retain protein disulfides in a reduced state. The effect of aggrelyte‐2 through acetylation might be long‐lasting because sirtuins, which can deacetylate proteins, are likely inactive in aged lenses, especially in the nuclear region. In addition, if sufficient NAD^+^ (a cofactor of sirtuins) is not available in the nuclei of lenses, it could further lower the ability of sirtuins to deacetylate proteins. Thus, aggrelyte‐2 may show superior long‐term effects on lens stiffness and might require fewer applications to obtain the desired results in the lens.

The mechanism of presbyopia is perhaps not entirely due to protein aggregation; studies involving mice show that other mechanisms could play a role. Individual proteins have been shown to contribute to lenses' biomechanical properties and stiffness. For example, the interaction between the lens fiber cell cytoskeletal intermediate protein CP49 and tropomodulin‐1 has been shown to control mechanical stiffness in mouse lenses (Gokhin et al., 2012). Ankyrin‐B in the plasma membrane, through its interactions with other cell adhesive proteins, has been shown to play a critical role in the fiber‐cell interaction and biomechanical properties of the lens (Rao & Maddala, 2016). Other proteins such as Dp71, aquaporin 0, and connexin‐50 have also been shown to be important for lens stiffness (Gu et al., 2019; Karnam et al., 2021). Whether these findings in mice are relevant to human presbyopia is unclear, but it is possible that chemical or structural modifications in these proteins could have effects on the biomechanical properties of the lens. Thus, targeting protein aggregation alone may not entirely rectify presbyopia in humans but could partially or largely restore the accommodative ability of the lens.

The lack of animal models for presbyopia hinders the evaluation of candidate compounds in live animals. Currently, the best approach involves testing compounds in cultured lenses or applying compounds topically to rodent eyes. However, in contrast to human lenses, rodent lenses do not accommodate; thus, any observed reduction in stiffness in rodents should be treated with caution. We interpret our observation of a decrease in stiffness in the lenses of mice as aggrelyte‐2 reduced stiffness through acetylation and disulfide reduction. Whether such effects restore/improve the accommodative ability of the lens cannot be tested in rodents. Nonetheless, in a future study, we plan to test whether aggrelyte‐2 topically applied to the eye can reach the lens and reduce stiffness in aged mice and rats. Such a study is important as the topical application might be a preferred and easier route (than intracameral application) to deliver drugs to the lens.

The ability of aggrelyte‐2 to reduce lens stiffness may not be entirely due to an increase in lens protein solubility. As mentioned above, an increase in lens stiffness can occur due to age‐related modifications in the zonules, lens capsule, and fiber cell membrane proteins. Our initial results (Figure S9) showed that the fiber cell architecture, at least in the cortical region of human lenses, was unaltered by aggrelyte‐2 treatment, suggesting that even if aggerlyte‐2 formed AcK modifications in the cell membrane, it did not grossly alter the fiber cell architecture under the conditions employed to test the effects in organ‐cultured human lenses. Further tests are required to ascertain whether acetylation of capsule, zonule, and plasma membrane proteins of fiber cells contribute to the reduction in lens stiffness.

Advanced glycation endproducts formed through the glycation of lysine and arginine residues in proteins accrue with age in human lenses and are predominantly present in the WI of lenses (Nagaraj, Linetsky, & Stitt, 2012). AGE formation has been considered a significant mechanism in lens protein aging and cataract formation. Age‐associated oxidative stress can accelerate lens protein glycation through ascorbate oxidation (Linetsky et al., 2014; Saxena et al., 2000). Acetylation blocks the free amine group of lysine residues in proteins making them unavailable for glycation. Therefore, the ability of aggrelyte‐2 to inhibit AGE formation through the acetylation of lysine residues could be another pathway by which it can prevent presbyopia. It has been shown that the binding of α‐crystallin in the lens to the fiber cell membrane increases during aging (Cobb & Petrash, 2000), and the ability of proteins to bind the membrane decreases upon lysine acetylation (Okada et al., 2021). Thus, the acetylation of proteins by aggrelyte‐2 could reduce their membrane binding during lens aging and inhibit presbyopia. In addition, protein deamidation is a major PTM in human lenses. Deamidation of β‐ and γ‐crystallins leads to their oxidation and aggregation (Norton‐Baker et al., 2022; Takata et al., 2008). Whether aggrelyte‐2‐mediated acetylation can reverse such protein aggregation could be investigated in a future study. A schematic illustration of the possible mechanisms by which aggrelyte‐2 might prevent presbyopia is shown in Figure S10.

Our observation of a reduction in stiffness by aggrelyte‐2 in aged human lenses is significant and relevant to presbyopia. The absence of toxic effects on lens epithelial cells and lens transparency are encouraging results and provide assurance that aggrelyte‐2 could be developed as a therapy against presbyopia. However, we would like to emphasize that our study provides a proof‐of‐concept demonstration and that compounds based on aggrelyte‐2 may have to be further refined for better lens penetrance and greater effects on reducing lens stiffness. We also recognize that further studies are warranted to determine whether treatment with aggrelyte‐2 causes any ultrastructural changes in lens components. In addition, testing in nonhuman primates might be required to determine its effects on lens accommodation.

## 4 EXPERIMENTAL PROCEDURE

### 4.1 Materials

N,S‐diacetyl‐L‐cysteine methyl ester (aggrelyte‐2) (Cat# D95910) was obtained from Astatech. N‐Acetyl‐L‐cysteine methyl ester (aggrelyte‐2C) (Cat# 01042) was purchased from Sigma–Aldrich. Monoclonal antibodies against AcK (Cat# 9681 S) and horseradish peroxidase (HRP)‐conjugated anti‐mouse IgG (Cat# 7076 S) were obtained from Cell Signaling Technology. All other chemicals were of analytical grade.

### 4.2 Stability of aggrelytes

Aggrelytes (2 mg each) were incubated in 1 mL of 50 mM phosphate buffer, pH 7.4, at 37°C. Aliquots were withdrawn immediately after incubation (Day 0) and after 1, 3, and 7 days. We tested the stability of the aggrelytes using ^1^H‐NMR (in DMSO‐d_6_) spectroscopy. The NMR peak integration values for the S‐acetyl or N‐acetyl cysteine methyl proton were compared with the cysteine methine proton.

### 4.3 Relative ability of aggrelyte‐2 to acetylate αAC

Human recombinant αAC (3 mg/mL PBS) was incubated for 24 h at 37°C while shaking without or with 500 μM acetylating agent (aggrelyte‐2, acetyl‐CoA, acetic anhydride or aspirin), dialyzed against PBS for 24 h at 4°C and digested with enzymes as described below. AcK in the digested samples was analyzed by LC–MS/MS, as described below.

### 4.4 Extraction of WI from human lenses

Human lenses (donor age: 55–75 years) were obtained from Saving Sight (Kansas City, MO), and Lions Eye Institute for Transplant & Research (Tampa, FL). The lenses were harvested within 36 h postmortem, stored with (when used for culturing) or without minimum essential medium (MEM), and shipped to our laboratory on ice or frozen on dry ice. Lenses shipped on dry ice (used in the solubility studies) were stored at −80°C until use. Each frozen lens was thawed on ice and homogenized with 1.5 mL of PBS in a hand‐held glass homogenizer. The homogenate was centrifuged at 20,000 *g* for 20 min at 4°C. The supernatant was discarded, and the pellet was suspended in 1 mL of homogenization buffer and centrifuged as described above. The resulting pellet was lyophilized and designated the WI fraction.

### 4.5 Incubation of WI with aggrelytes and estimation of the solubilized protein content

Stock solutions (10 mM) of aggrelyte‐2 and aggrelyte‐2C were prepared in 50 mM phosphate buffer. WI (2 mg) was suspended in 0.4 mL of 50 mM sodium phosphate buffer, pH 7.4. Aggrelyte‐2 or aggrelyte‐2C was added to the suspension to obtain a final concentration of 50–2000 μM. The mixture was incubated at 37°C while shaking for 24 or 48 h after adding 0.002% sodium azide to prevent bacterial growth. After incubation, the samples were centrifuged at 20,000 *g* for 20 min at 4°C, and the supernatants were collected. We measured the protein in the supernatant using a BCA Protein Assay Kit from Thermo Scientific using BSA as the standard. The lack of reaction with BCA in the filtrate suggested that aggrelytes did not interfere in the BCA assay.

### 4.6 Western blot analysis of AcK in solubilized protein

Proteins were separated on a 12% denaturing gel, transferred to a nitrocellulose membrane, blocked with 5% nonfat dry milk, and incubated with the AcK antibody (1:5000 dilution) for 16 h, followed by incubation with HRP‐conjugated goat anti‐mouse IgG (1:5000 dilution) for 1 h. Chemiluminescence was detected using an Enhanced Chemiluminescence Detection Kit (Thermo Scientific). Then, the membrane was stained with Ponceau‐S to visualize the proteins and normalize the detected protein to the total protein applied.

### 4.7 Identification of crystallins in the solubilized protein

We performed western blot analyses of the solubilized protein of human lenses (after treatment with or without aggrelytes as described above) using antibodies against αAC (Enzo Life Sciences, Cat# ADI‐SPA‐221D, dilution 1:14,000), αBC (the University of Iowa, dilution 1:700,000), βC (Santa Cruz Biotechnology, Cat# sc‐22745, dilution 1:14,000) and γC (Santa Cruz Biotechnology, Cat# sc‐22746, dilution 1:7000). The secondary antibodies were HRP‐conjugated anti‐mouse IgG for αBC and HRP‐conjugated rabbit IgG (Cat# 7074 S, Cell Signaling Technology) for αAC, βC, and γC. According to the supplier, the βC antibody detects mostly βB1‐crystallin and to a lesser extent, βA1/3, βA2, βA4, βB2, and βB3‐crystallin. The γC antibody reacts with γA, γB, γC, γD, γE and γF‐crystallin. The other details of the western blot analyses were as described above.

### 4.8 LC–MS/MS measurement of AcK

Solubilized WI protein (0.5 mg/mL) fractions and WS proteins (1 mg/mL from mouse lenses and 3 mg/mL from human lenses) were sequentially digested with enzymes as previously described (Nandi et al., 2019). The enzyme‐digested samples were analyzed for AcK by LC–MS/MS using the standard addition method, as previously described (Nandi et al., 2019). The AcK levels were calculated based on the protein input in the enzymatic digestions.

### 4.9 Protein‐thiol estimation

Immediately after the treatment with aggrelytes (24 h), 0.15 mL samples were filtered through 10 kDa cutoff centrifugal filters at 4°C. Centrifugal filtration was repeated twice by adding 0.4 mL of 50 mM N_2_‐bubbled sodium phosphate buffer to the retentate each time. Ten micrograms of protein from the final retentate was used for thiol content estimation using a Thiol Quantification Assay Kit (Cat# ab112158, Abcam) using reduced GSH as the standard.

### 4.10 Cell cytotoxicity

Mouse lens epithelial cells (primary cells from lenses of 1‐month‐old C57BL/6J mice, passages 3–5) were incubated with aggrelyte‐2 or aggrelyte‐2C at concentrations from 0–1000 μM for 24 h. Primary human lens epithelial cells (isolated from a 47‐year‐old donor, passages 3–5) were incubated with 0–1000 μM aggrelyte‐2 or aggrelyte‐2C for 72 h with a change in the media containing freshly dissolved aggrelyte every 24 h. Cell viability was measured by MTT assay.

### 4.11 Effect of aggrelytes on lens stiffness, protein solubility, and acetylation

All animal experiments were reviewed and approved by the Institutional Animal Care and Use Committee (IACUC) of the University of Colorado, Aurora and performed in adherence to the ARVO Statement for the Use of Animals in Ophthalmic and Vision Research. Mice (C57BL/6J, 5–8 months old, Jackson Laboratories, Barr Harbor, ME, Stock No. 000664) were used in the study. The lenses were dissected from the eyes by a posterior approach and incubated with or without aggrelyte (1 mM) for 24 h in serum‐free and phenol‐red‐free MEM at 37°C. The osmolarity of the medium was adjusted to 297 mOsm by the addition of cell culture grade H_2_O.

The stiffness of freshly obtained lenses was measured as previously described (Baradia et al., 2010). Briefly, the lens was placed on a flat platform with the anterior side facing up. The load was applied to the top of the lens at 100 mg increments in the case of mouse lenses and 250 mg increments in the case of human lenses. The displacement in the lens axial diameter (in μM) was measured using MATLAB software developed by Dr. Adrian Glasser, UK, and plotted against the load applied. The displacement at a specific load was used to determine differences in lens stiffness between the untreated control and aggrelyte‐treated lenses. The axial strain of lenses was calculated based on the following formula:

$$\frac{\left(\text{Diameter of the lens after the load applied}-\text{initial diameter of the lens}\right)}{\text{Initial diameter of the lens}}$$

Human lenses (42–68 years of age) were incubated with or without aggrelyte‐2 (1 mM) for a total of 72 h in phenol red‐free MEM with a change in media containing freshly dissolved aggrelytes every 24 h. One lens from each donor pair was used as the control, and the other was treated with aggrelyte. After incubation, the lens stiffness was measured, and the lenses were decapsulated, transferred to a 5‐ or 10‐mL round bottom flask containing 1 mL of 50 mM phosphate buffer, pH 7.4, and stirred using a magnetic stir bar to solubilize the protein from the cortex of the lens. The remaining nucleus was homogenized in 1 mL of phosphate buffer. Both cortical and nuclear protein extracts were centrifuged at 20,000 *g* for 20 min at 4°C to obtain the soluble protein in the supernatant. Western blot analysis of AcK‐bearing proteins was performed using 10–20 μg of protein from each lens sample as described above. In the case of mouse lenses, water‐soluble protein (WS) was obtained by homogenizing each mouse lens in 0.2 mL of PBS, followed by centrifugation at 20,000 *g* for 20 min at 4°C. The AcK content was measured as described above.

In another set of experiments, human lenses (from donors aged 42–64 years) were incubated with aggrelytes as above. After incubation, each lens was washed with PBS and homogenized using 1.5 mL 50 mM phosphate buffer, pH 7.4. The homogenate was centrifuged at 20,000 *g* for 20 min to obtain a supernatant. We measured the protein in the supernatant to determine the effect of aggrelyte treatment on the soluble protein content in the lens.

### 4.12 Mass spectrometry of solubilized proteins to determine the AcK site‐specific levels

Three hundred fifteen micrograms of cortical protein from the aggrelyte‐2‐treated (49‐year‐old lens) (as above) and untreated control human lenses were prepared as previously reported for AcK site‐specific analysis by LC/MS and LC–MS/MS (Nandi et al., 2019). Briefly, samples were spiked with 200 ng of AcBSA standard, reduced, alkylated, and digested with trypsin using a urea‐based method and then desalted using a C18 resin before being lyophilized. The samples were then immunoprecipitated according to the manufacturer's protocol for the PTMScan Acetyl‐Lysine Motif Kit (Cat# 13416, Cell Signaling Technology). Peptides were purified with a Pierce C18 spin column (Thermo Fisher Scientific Inc.) and dried in a Speed Vac concentrator at 45°C. The peptides were resuspended in 3% acetonitrile in 0.1% formic acid for MS analysis.

The enriched AcK‐bearing peptides were loaded onto a 2 cm PepMap 100 nanoviper trapping column and chromatographically resolved with a 0.075 × 250 mm, 2.0 μm Acclaim PepMap RSLC reversed‐phase nanocolumn (Thermo Scientific) using a 1290 Infinity II LC system equipped with a nanoadapter (Agilent). The mobile phase consisted of water +0.1% formic acid (A) and 90% aqueous acetonitrile +0.1% formic acid (B). Samples were loaded onto the trapping column at 3.0 μL/min for 3.5 min under initial conditions before being chromatographically separated at an effective flow rate of 330 nL/min using a gradient of 3–8% B over 3 min, 8%–30% B over 33 min, and 30%–40% B over 4 min at 40°C. The gradient was followed by a column wash at 75% B for 5 min. Data were collected using a 6550 Q‐TOF equipped with a nanosource (Agilent Technologies) operated using intensity‐dependent CID MS/MS to generate peptide identifications. MS/MS data were collected in positive ion polarity over a mass range of 290–1700 m/z at a scan rate of 8 spectra/second for MS scans and a mass range of 50–1700 m/z at a scan rate of 3 spectra/second for MS/MS scans. All charge states, except singly charged species, were allowed during MS/MS acquisition.

Tandem mass spectra were extracted, searched, and summarized using PEAKS X+ Studio 10.5 software (Bioinformatics Solutions Inc.). The spectra were searched against the UniProtKB SwissProt [2022_02] Homo sapiens database, allowing for up to three missed semitryptic cleavages with fixed carbamidomethylation of cysteine and a maximum of four variable modifications of deamidation of asparagine and glutamine, oxidation of methionine and/or acetylation of lysine per peptide. The allowed monoisotopic peptide mass tolerance was ±15.0 ppm, and the MS/MS tolerance was ±0.05 da. A FDR filter of 0.5% was used corresponding to a –10logP score ≥ 19.3.

The MS‐only level data from the LC–MS/MS analyses were extracted and aligned using Profinder V.B.10.01 software (Agilent Technologies). Retention times, neutral masses, and chemical formulas generated from identified acetyl peptides were used to perform batch‐targeted feature extraction. Data were extracted with an ion count threshold of two or more ions, 5000 counts, and a score threshold of 70. The score was based on the quality of the mass accuracy, isotope abundances, and isotope spacing of compounds based on the chemical formula of the target peptides within a specified retention time window. Using the peptide isotope model, charge states 2–6 were allowed with H^+^ and Na^+^ charge carriers. The retention time window and mass window alignment tolerances were set to 0.4 min and 10 ppm, respectively. The extracted peptide peak areas were then used to determine a fold change value in the treated vs. control samples to help identify potential AcK sites that may be affected by aggrelyte‐2. Due to a lack of statistical power, a three‐fold change filter was used as a sufficient cutoff to identify potential AcK sites of interest. The list of AcK sites that passed the three‐fold change cutoff was further curated to include only those AcK sites for which all corresponding peptides with the same AcK site had a three‐fold change or greater when summed and averaged together.

Samples were also searched using the UniProtKB SwissProt Bos taurus database to identify peptides from the acetylated BSA standard spiked‐in at the beginning of sample preparation. All other search and MS‐only level extraction parameters were unchanged compared to the SwissProt Homo sapiens workflow. Extracted acetylated BSA peptide peak areas were summed and used to determine a fold change value in treated versus control samples.

### 4.13 Effects of aggrelytes on lens morphology

After incubation with or without 1 mM aggrelytes for 72 h, the human lenses were fixed in 1% paraformaldehyde/1.25% glutaraldehyde in 0.08 M sodium cacodylate buffer, pH 7.4, for 5 days at 4 °C, paraffin‐embedded, and sectioned (4 μm). After deparaffinization, the sections were stained with lectin‐FITC (Dilution 1:100, Millipore Sigma, L4895) in 1% BSA (Millipore Sigma, A1498) and 0.3% Triton X‐100 (Across Organics) in PBS overnight at 4 °C. The slides were then washed and counterstained with Vectashield (Cat# H‐1200, Vector Laboratories, Inc.) containing DAPI. The slides were imaged under a Nikon Eclipse Ti confocal microscope (Nikon).

### 4.14 Effect of aggrelyte‐2 on AGE formation

Human recombinant αAC (3 mg/mL) or γD‐crystallin (γDC, 2 mg/mL) in PBS was treated with or without aggrelyte‐2 (10 mM for αAC and 1.25 mM for γDC) for 24 h, dialyzed against 100 mM sodium phosphate buffer pH 7.4 for 16 h and incubated with or without a glycating mixture (2 mM ascorbate, 25 mM D‐glucose and 250 μM methylglyoxal) in 100 mM sodium phosphate buffer pH 7.4 for 5 days (after adding 0.002% sodium azide) at 37°C. The incubated proteins were dialyzed against PBS for 16 h, treated with 100 mM sodium borohydride for 1 h at room temperature, and acid hydrolyzed, and CML and CEL were analyzed by LC–MS/MS as described above.

### 4.15 Statistics

All data are expressed as the mean ± standard deviation (SD). The experiments were performed with at least triplicate samples. Comparisons between two groups were performed by paired or unpaired Student's *t* test, and comparisons among multiple groups were performed by one‐way ANOVA with Tukey's multiple comparison test.

## AUTHOR CONTRIBUTIONS

RHN conceived the project. RHN, RBN, SP, MN, JR, HG, and CRM designed the experiments; SP, RBN, JR, CRM, HG, and MN conducted the experiments; RHN, SP, RBN, JR, CRM, HG, and MN performed data analysis. RHN, SP, RBN, JR, CRM, HG, and MN wrote the manuscript. RHN supervised the project. All authors have approved the final version of the manuscript for publication.

## CONFLICT OF INTEREST STATEMENT

The authors declare that they have no conflicts of interest with the contents of the article.

## Supporting information


Appendix S1.

## Data Availability

All data generated or analyzed during this study are included in the article. The mass spectrometry proteomics data have been deposited to the ProteomeXchange Consortium via the PRIDE (Perez‐Riverol et al., 2022) partner repository with the dataset identifier PXD035049.
