# Supplementary material for: Aggrelyte‐2 promotes protein solubility and decreases lens stiffness through lysine acetylation and disulfide reduction: Implications for treating presbyopia
Source: Aging Cell. 2023 Feb 23;22(4):e13797. doi: 10.1111/acel.13797 (PMC10086532; doi:10.1111/acel.13797)
Supplement: Supplementary file 1 — Appendix S1. [file ACEL-22-e13797-s001.docx]

**Figure S1**

**Figure S1. Effect of aggrelyte concentration on protein solubility.** WI (2 mg/0.4 mL) from a human lens (71 years) was suspended in 50 mM phosphate buffer, pH 7.4. To the suspension, aggrelyte-2 or aggrelyte-2C (0-2000 µM) was added and incubated for 48 h at 37 °C. The suspension was centrifuged at 20,000 × g for 20 min, and the soluble protein content in the supernatant was measured. At each concentration, each sample was separately processed three times and analyzed. The data are the mean ± S.D. for each sample processed three times separately and analyzed. Statistical comparisons between the controls and treated samples were performed. *p<0.05, ***p<0.001, ****p<0.0001, ns=not significant

**Figure S2**

**Figure S2.** **Aggrelytes solubilize human lens WI.** WI (2 mg/0.4 mL) from aged lenses (65-75 years) was suspended in 50 mM phosphate buffer, pH 7.4, and treated with 500 µM of one of the aggrelytes for 48 h **(A)**. Each sample was separately processed three times and analyzed (mean ± S.D.). The combined effects of the aggrelytes (from 10 lenses) on the solubilization of WI protein are shown in Panel **B**. The percent solubilized protein from the initial weight of WI after 48 h treatments is shown in Panel **C**. *p<0.05, ***p<0.001, ****p<0.0001.

**Figure S3**

**Figure S3. Aggrelyte-mediated solubilization of WI is not due to the release of soluble proteins adherent to WI:** Four-times-washed WI (2 mg/0.4 mL) from an aged lens (67 years) was suspended in 50 mM phosphate buffer, pH 7.4, and treated with 500 µM of one of the aggrelytes for 24 h. Each sample was separately processed three times, and the soluble protein content was determined by BCA assay. The bar graphs represent the mean ± S.D. of measurements. **p<0.01, ***p<0.001, ns=not significant

**Figure S4**

**Figure S4. Protein solubility was better in aggrelyte-2-treated than acetic anhydride-treated human WI.** WI (2 mg/0.4 mL) from a 73-year donor lens was suspended in 50 mM phosphate buffer, pH 7.4, and treated with 500 µM of one of the aggrelytes or acetic anhydride for 24 h (**A**). Each sample was separately processed three times, and the soluble protein content was determined as above. A western blot image shows the AcK levels in 5 µg of solubilized samples **(B)**. The Ponceau S-stained membrane shows equal protein loading **(C)**. A densitometric plot from the western blot analysis of the AcK-bearing proteins is shown in **(D)**. The bar graphs represent the mean ± S.D. of measurements. **p<0.01, ***p<0.001, and ****p<0.0001.

**Figure S5**


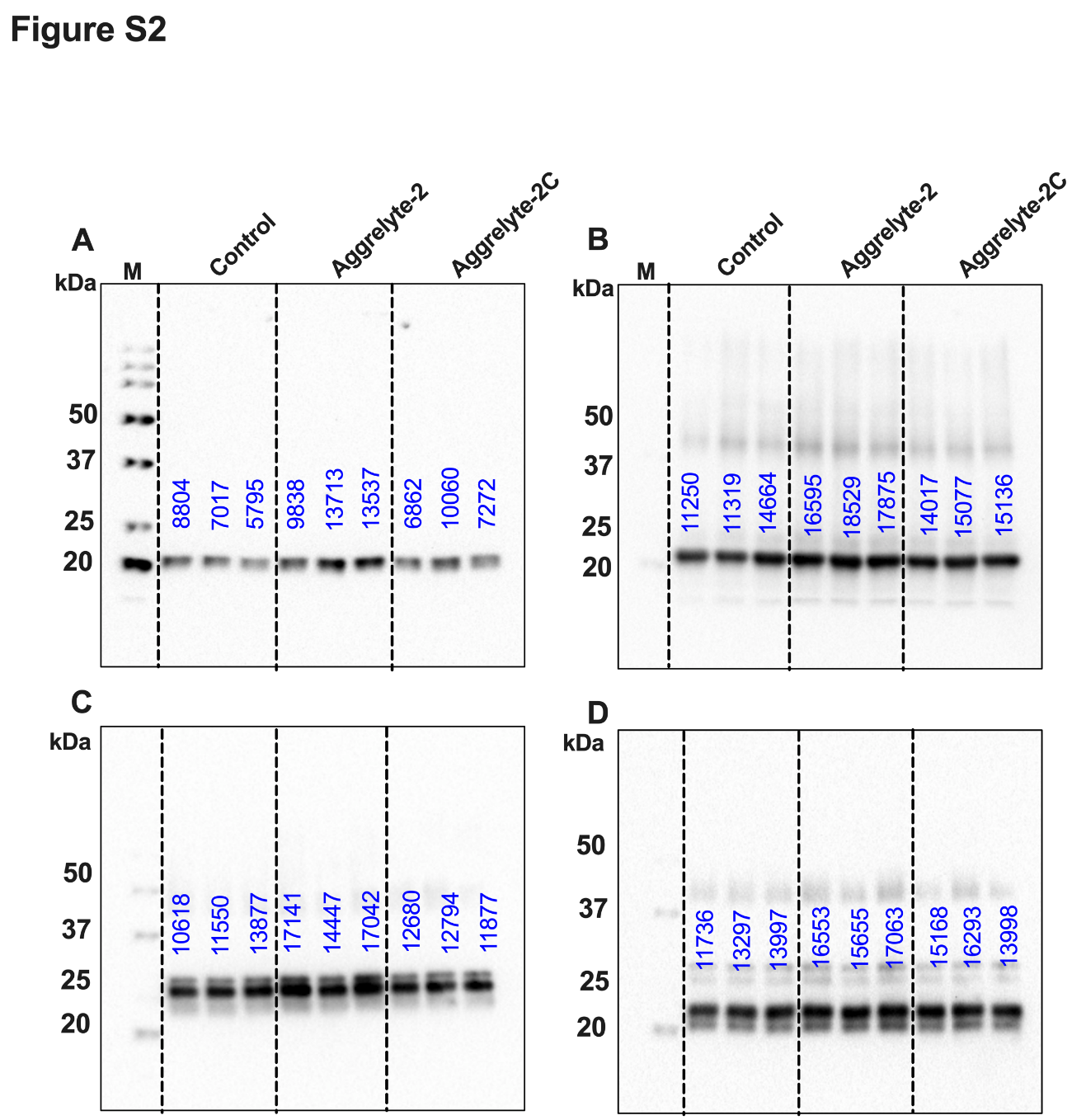


**Figure S5. Individual crystallin levels in the solubilized protein.** Representative western blots for αAC **(A),** αBC **(B**), βC **(C)** and γC **(D)** are shown for the aggrelyte-2-solubilized protein from a 73-year-old lens treated with aggrelyte-2 or aggrelyte-2C (each 500 µM), as described in Figure 2. Each sample was separately processed three times and analyzed. The densitometric value for each protein band is shown.

**Figure S6**

**Figure S6. Effects of aggrelytes on lens epithelial cell viability.** Primary mouse lens epithelial cells (isolated from 1-month-old C57Bl6/J mice, passage 3-5) were treated with aggrelytes for 24 h **(A)**. Primary human lens epithelial cells (isolated from a 47-year-old noncataractous lens, passage 3-5) were treated with aggrelytes for a total of 72 h, with a change in media containing freshly dissolved aggrelytes every 24 h **(B)**. Cell viability was tested by MTT assay.

**Figure S7**

**Figure S7. Aggrelytes do not change the transparency and weight of human lenses.** Representative images of two sets (set 1=A and B, 60 years, and set 2=C and D, 62 years) are shown. Lenses were incubated without (control) or with aggrelyte-2 or 2C (1 mM) in serum-free MEM for 72 h as described in Methods. The images show transparency of the control (**A** and **C**) and aggrelyte-2 (**B**) or 2C (**D**) treated lenses. The weight of lenses was measured before and after incubation, and the percentage change in weight was calculated (**E** and **F**). ns=not significant.

**Figure S8**

**Figure S8. Aggrelyte-2 reduces the axial strain of mouse and human lenses.** Lenses were incubated without (control) or with aggrelyte-2 or 2C (1 mM) in serum-free MEM as described in Methods. The bar graph for changes in the axial strain of mouse lenses is shown at a 100 mg load **(A)**. The changes in the axial strain of human lenses treated with aggrelyte-2 at loads of 500 and 1000 mg are shown in **(B),** and changes in axial strain for human lenses treated with aggrelyte-2C at loads of 500 and 1000 mg are shown in **(C)**. Each data point represents a value for an individual lens. *p<0.05, **p<0.01, ns=not significant.

**Figure S9**

Aggrelyte-2C

Aggrelyte-2

Control


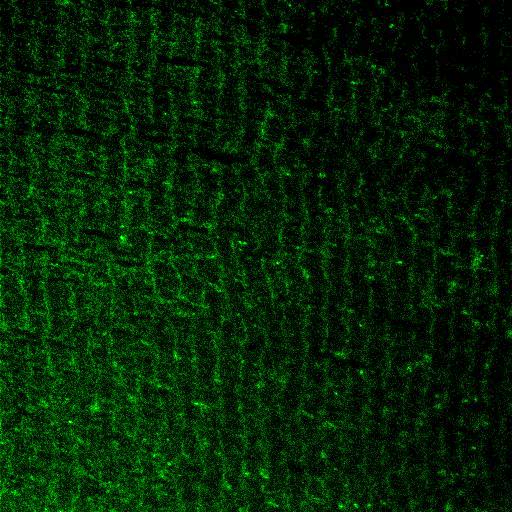

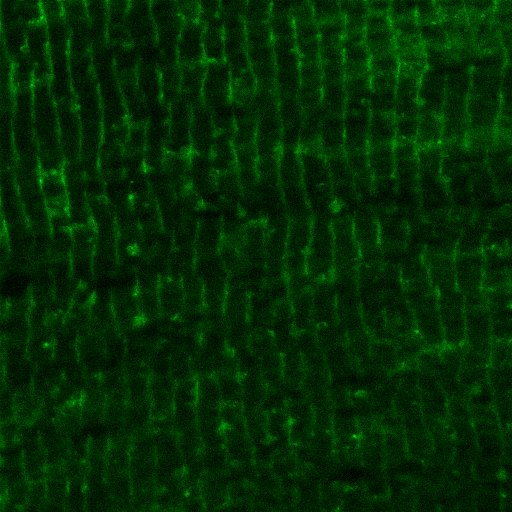

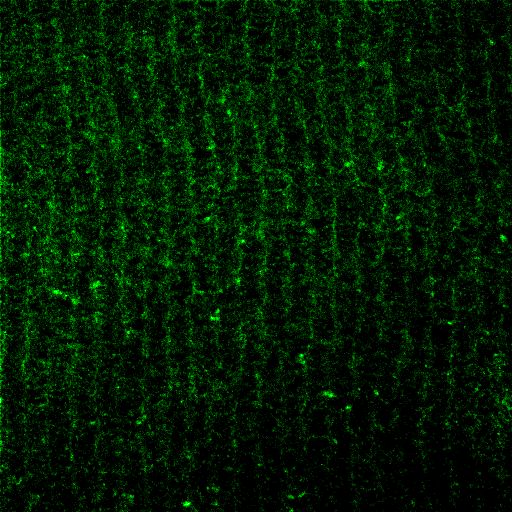


Lectin-FITC

Scale bar = 50 μm

**Figure S9. Effect of the aggrelytes on the morphology of human lenses.** Fluorescence images of lectin-FITC-stained outer cortical sections of a 63-year-old human lens treated with aggrelyte-2 or aggrelyte-2C (1000 μM for 72 h).

**Figure S10**


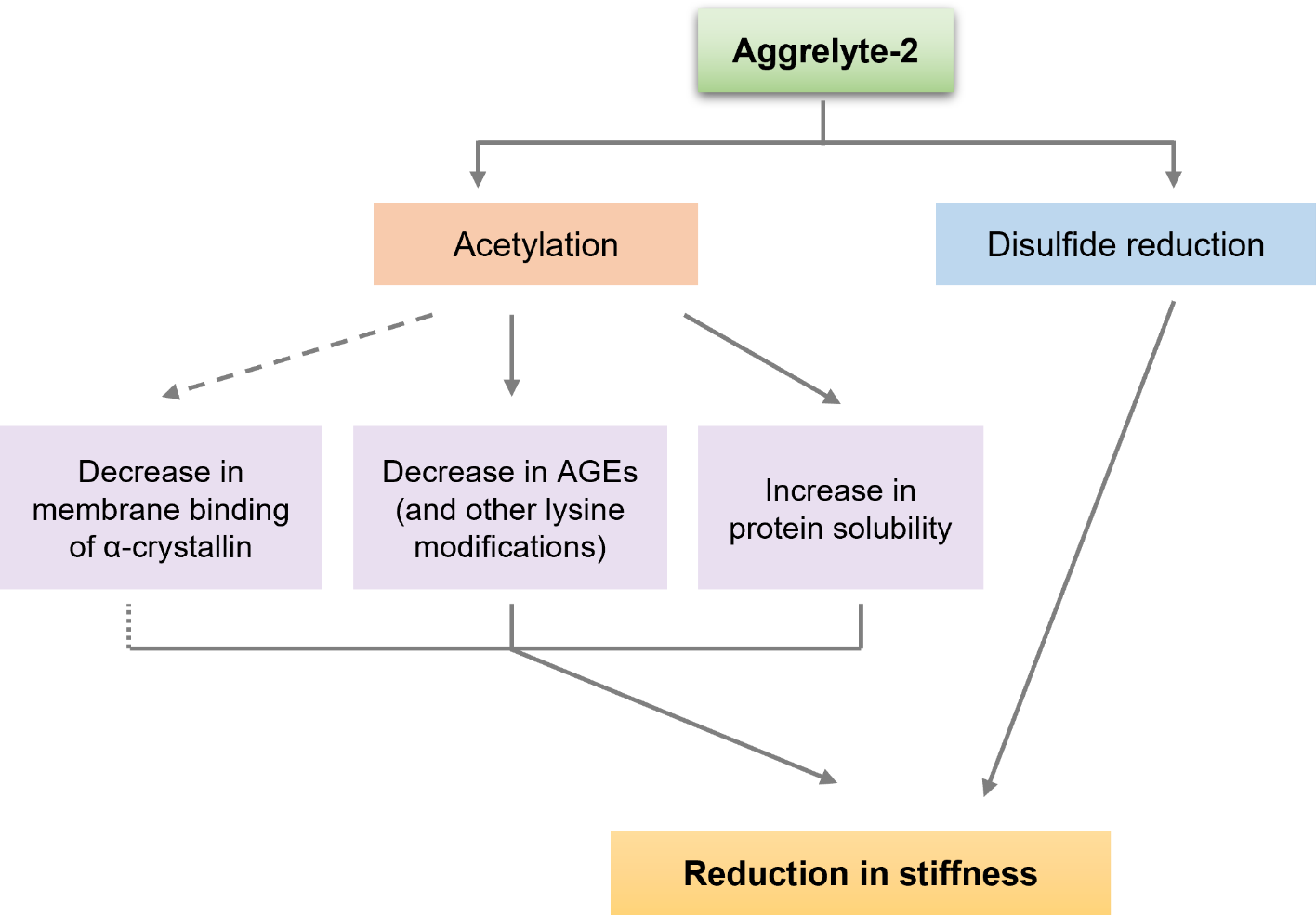


**Figure S10. Possible mechanistic pathways by which aggrelyte-2 increases protein solubility and decreases stiffness in aged lenses.** It is possible that acetylated proteins bind less to the lens fiber cell plasma membrane (dashed line) during aging.

| Time (day) | Aggrelyte-2 | | | Aggrelyte-2C | | |
| --- | --- | --- | --- | --- | --- | --- |
|  | S-acetyl methyl proton | Methine proton* | Stability (%) | N-acetyl methyl proton | Methine proton* | Stability (%) |
| 0 | 3.00 | 1.09 | 100 | 3.00 | 0.85 | 100 |
| 1 | 3.00 | 1.17 | 93 | 3.00 | 1.00 | 85 |
| 3 | 3.00 | 1.56 | 70 | 3.00 | 1.08 | 79 |
| 7 | 3.00 | 1.87 | 58 | 3.00 | 1.34 | 63 |

**Table S1.** **Stability of aggrelyte-2 and aggrelyte-2C**

* The methine proton integration value was calculated by combining the integration values before and after methyl ester desertification.
